# Local government interventions for improving the health and wellbeing of tenants in private rented housing: developing initial program theory to inform evaluation in the United Kingdom

**DOI:** 10.1186/s12889-024-19163-9

**Published:** 2024-08-07

**Authors:** Rachael McClatchey, Claire F. Ferraro, Ellis Turner, Jennifer Harris, Jonathan Banks

**Affiliations:** 1 School of Health and Social Wellbeing, University of the West of England, Coldharbour Lane, Stoke Gifford, Bristol, BS16 1QY UK; 2 Office for Health Improvement and Disparities, Department for Health and Social Care, Temple Quay, 2 Rivergate, Bristol, BS1 6EH UK; 3 School for Policy Studies, University of Bristol, 8 Priory Road, Bristol, BS8 1TZ UK; 4 National Public Health Speciality Training Programme, South West, Bristol, UK; 5 Tenancy Deposit Scheme, First Floor, West WingThe Maylands Building, 200 Maylands Avenue, Hemel Hempstead, Hertfordshire, HP2 7TG UK; 6 Applied Research Collaborative West, National Institute for Health and Care Research, University Hospitals Bristol and Weston NHS Foundation Trust, Bristol, BS21 2NT UK; 7 Bristol Medical School, Population Health Science, University of Bristol, Bristol, BS8 1QU UK

**Keywords:** Private Rented Housing, Public health, Realist evaluation, Causal mechanisms, Local Government

## Abstract

**Background:**

Housing is an important wider determinant of health. Private Rented Sector (PRS) housing is generally the worst quality of housing stock across tenures. Although a wide range of interventions are available to local governments to manage and improve the quality of PRS housing and therefore the health of tenants, there is limited evidence about the extent to which these are used. This study aims to explore what drives the use of different interventions in different local governments, to better understand and inform local strategies.

**Methods:**

As the first realist evaluation on this topic, the range of available interventions was informed by a Local Government Association toolkit. Consistent with realist approaches, retroductive analysis of intervention-context-mechanism-outcome configurations helped to develop and refine Initial Programme Theories (IPTs). Data sources included local government housing documents, a survey and eleven semi-structured interviews with housing officers.

**Results:**

Using data for 22 out of the 30 local governments in the South West region of the United Kingdom, eight IPTs were developed which act on different levels from individual PRS team leaders to system wide. The IPTs include a belief in market forces, risk adverse to legal challenge, attitude to enforcement, relational approaches to partnership working, job security and renumeration, financial incentives drive action, and system-level understanding of the drivers of poor health, inequalities and opportunities for cost-savings. The findings suggest that limited objective health outcomes are being used to understand impact, which hinders interpretation of the effectiveness of all mechanisms.

**Conclusion:**

Interventions that bring about positive outcomes in managing PRS housing are unlikely to be universal; they depend on the context which differs across place and over time. The proposed IPTs highlight the need for strategies to be tailored considering the local context and should be evaluated in subsequent phases of study.

**Supplementary Information:**

The online version contains supplementary material available at 10.1186/s12889-024-19163-9.

## Introduction

There is extensive evidence demonstrating the importance of housing as a wider determinant of health, and of inequalities in health [1, 2]. There are interlinking pathways through which housing impacts on numerous health outcomes including cardiorespiratory diseases, infectious diseases, injuries, allergies and mental health conditions [1–4]. Despite a substantial evidence base showing which features of housing are beneficial or harmful to health, 1.6 billion people, or 20 per cent of the world’s population, live in inadequate, crowded and unsafe housing [5]. This has significant implications on occupants lives and for wider health and social care systems. In the United Kingdom (UK), it is estimated that the National Health Service (NHS) spends £2.5billion/year on housing and health-related conditions (e.g., primary care visits, prescriptions, and hospital treatment), and £18.5billion/year on wider societal costs, such as those relating to care [6]. This suggests that the quality of people’s housing has a similar impact on health as does smoking (£2.3–3.3billion/year) or alcohol consumption (£3.2billion/year) [7].

Private Rented Sector (PRS) housing is generally the worst quality of housing stock across tenures. For example, in England, 14% of all homes are classified as non-decent; and this figure rises to 23% in the PRS [8]. Studies have reported that people living in PRS housing are more likely to experience poor mental health, have higher levels of a stress biomarker and show higher mortality rates compared to homeowners [9, 10]. Explanations for this include issues relating to affordability, landlord/tenant relationships and tenure insecurity [11]. The proportion of households which are rented varies internationally, from 5% in Romania to 58% in Switzerland [12]. In recent years the PRS has experienced significant growth in some countries, doubling over 20 years, from 10% of British households in 2000, to 19% in 2021/22 [8]. Tenancy lengths are increasing and families with children are remaining in the sector for longer [11].

### Wide range of available interventions

In the PRS, a wide range of interventions are available to local governments to manage and improve the quality of PRS housing and therefore the health of tenants, which are collated in the ‘Improving the PRS: A toolkit for councils’ (LGA toolkit) [13]. This was produced by the Housing Quality Network for the Local Government Association and was informed by a policy and research review, interviews with national stakeholders, and case studies [14].

A key characteristic of PRS housing is its complexity. Types of landlords can include temporary or accidental landlords, individuals with property portfolios and institutional investors. Demand is driven by a diverse range of groups including key workers, students, households unable to access owner occupation or social housing, migrant workers, young people on low incomes and homeless and vulnerable households [14, 15]. Local authorities face significant challenges in implementing interventions in practice, and approaches to regulating the sector vary significantly between localities [16]. Available interventions often involve multiple parties (such as tenants, landlords and housing officers), will occur across diverse and rapidly changing policy and funding landscapes (e.g. the Renters (Reform) Bill currently going through parliament in the UK), and can lead to wide-ranging outcomes and unidentified consequences [17, 18]. There is limited evidence on the extent to which available interventions are used, and what factors affect local governments in doing so. Recent evidence reviews on housing and health inequalities concluded that there is an urgent need for research to explore effective interventions in the PRS [19], which takes a holistic approach and can understand the complex pathways to outcomes [1].

### Realist methodology justification

Although knowledge about the pathways through which wider determinants influence health has accumulated and is growing, the complexity that is involved with studying and understanding these determinants, the exact pathways through which they operate, and how they affect population health outcomes continue to pose real challenges for advancing the field, and for developing coherent, practical interventions [20]. Progress on the provision of healthy privately rented housing is therefore likely to be most effective if the complexity is acknowledged and interventions are not considered in isolation [20, 21]. Although the application of methodological reductionism has been useful for answering certain causal questions about individual level, simple interventions, methods underpinned by complex systems theory are imperative for interventions on wider determinants of health [21]. Realist methodology is therefore becoming an increasingly popular way to synthesise complex public health interventions, such as those on healthy housing, as it allows a greater understanding of the intervention process, rather than simply deducing whether an intervention is effective or not [22]. Realist methods are particularly appropriate for evaluating programmes that produce mixed outcomes and to better understand ‘what works, for whom, in what circumstances’ [23].

To our knowledge there are no previous realist studies on delivering healthy PRS housing. Rolfe et al*.* (2020) used a realist approach [22], but this was to understand how housing acts as a determinant of health, rather than how healthier housing can be delivered in practice. Therefore, this study uses a realist evaluation, to understand what drives different interventions in different local governments in the hope of better understanding and informing local strategies.

### Aim

This exploratory study describes the development of Initial Programme Theories (IPTs) to provide insight into the factors that influence local government interventions to manage the quality of PRS housing and therefore the health and wellbeing of tenants.

Set in the South West (SW) region of the UK, these IPTs should be evaluated in subsequent phases of study, to inform the development of local government interventions to improve tenants’ health across the UK and beyond.

## Methods

### Initial programme theories

A realist approach allows evaluators to draw on a range of data sources to identify the important mechanisms and contextual factors that contribute to whether and how outcomes are achieved. These are captured in intervention-context-mechanism-outcome configurations (ICMOCs) [23]. Together, the ICMOCs make up a programme theory, which highlights the configurations needed for an approach to work. The development of IPTs for realist evaluation can occur through a variety of approaches including: realist synthesis of existing literature, further development of an existing program theory, qualitative research (such as, program documentation review, interviews, etc.) and/or through the experiential or professional knowledge of the research team [24, 25].

Given the nascent nature of the realist evidence base on housing and health [22], the latter two approaches have been adopted in this study. As the evidence base states that positive change is most likely if interventions on complex public health topics are not considered in isolation, [20, 21] the entirety of interventions listed in the LGA toolkit were considered in-scope of this study [14]. Table 1 shows how the IPTs were produced in phases, following the approach set out by Gilmore et al. [26].

**Table 1:** Summary of steps to develop and refine Initial Programme Theories (IPTs)

Stage of consultation	Source of expertise	Date
Early iteration of IPTs	Local Government Association ‘Improving the PRS: A toolkit for councils’ [13], housing documents and after five of the eleven interviews had been completed	October—November 2022
Discussion of IPTs	Research team	November 2022
Refinement following analysis of document, survey and interview data	Housing documents, survey and all eleven interviews	November 2022 – March 2023
Refinement of IPTs through experiential and professional experience	Research team	April 2023
IPTs finalised for testing	Research team including additional member (JB)	April–May 2024

### Recruitment and setting

Realist evaluations resist the notion of generalisability and give more value to exploratory theories about how interventions are shaped by context [23, 26]. The SW region was chosen as the setting because the PRS as a proportion of the overall housing stock is similar in the SW (19.4%) to the England average (19.5%) [8] and there is a mixture or rural and urban localities, and size and structure of local governments (e.g. single-tier and two-tiers, location of PRS team within council) to facilitate the exploration of different contexts.

In September 2022, using contacts from the research teams’ professional networks, a personalised email was sent to the PRS team leader in each single-tier and all districts within two-tier local authorities (as housing teams exist in each lower-tier local authority (LTLAs)) in the SW to complete an online survey [27]. In addition, each single-tier LTLA and a single district within each two-tier authority, were invited to take part in an interview by email and/or phone. Purposive sampling of which district to approach was based on the research team’s professional experience of where there were examples of innovative work in the PRS.

### Data collection

Three data sources collected between October and November 2022 were included: a survey, semi-structured in-depth interviews and a review of local government housing documents.

A survey, based on the LGA toolkit [13], was redesigned and abbreviated in Qualtrics software by the research team (Additional File 1). Whilst data on the LTLA respondents job grade and title was collected, individual survey respondents remained anonymous. Multiple responses from the same LTLA were encouraged to assess differences in responses between different cadre of staff. This enabled the profiling of LTLAs and the breadth of interventions being used in the SW to be understood.

An interview topic guide was developed to explore survey questions in-depth, with a particular focus on elucidating information on enablers and limitations in the use of different interventions. Questions addressing each theme in the LGA toolkit were included: evidence base, policy and policy making, resources, governance, partnerships, consumer regulation, and emerging issues [13] (Additional File 2). A pilot interview was conducted with a PRS team leader from a LTLA external to the SW. As per realist methodology, the topic guide was reviewed and updated with the research team after the first interviews [24, 26]. Each interview was recorded (video and audio) on Microsoft Teams. The automatic transcript was reviewed and cleaned immediately following the interview.

Housing documents (of any type, tenure, date) were downloaded from LTLA websites or requested from interviewees. These were summarised by type and date in Excel (Version 2210 16.0.1) with sections of text referring to strategic aims and action plans. This spreadsheet was then uploaded and coded in Nvivo. Additional housing information on webpages only were excluded.

### Data sources

A total of 26 survey responses from 18 unique LTLAs were received. LTLA duplicates were reviewed and the least complete response removed. Notable discrepancies existed between duplicate responses from different cadre of staff from Enforcement Officer, PRS team leader to Head of Environmental Health, where estimates of the number of full-time equivalent (FTE) staff working on the PRS varied five-fold. 10/18 survey respondents answered all or nearly all questions within the survey with notable drop-off following Q3.6 (Additional File 1).

Seventy six documents were included from 20 LTLAs, including at least one district from each two-tier local government. The main types of documents were housing strategies (n = 36), enforcement policies, housing stock and other evidence reports, strategic action plans, and guides for tenants and landlords. The housing strategies for each LTLA varied from overall strategies to more specific topics such as PRS, homelessness prevention (most common), social housing or accommodation with care and support. Timeframes for strategies varied with the majority of LTLAs having at least one housing strategy in-date (up to or including 2022).

Eleven interviews were conducted with ten unique LTLAs; one LTLA had a second interview with the same interviewee due to not completing the topic guide questions within the initial allotted hour. A single co-author (CFF) attended all interviews and conducted 10/11, whilst LD attended 8/11 and conducted one. Eight interviewees were Private Sector or PRS team leaders and two were PRS enforcement officers. The interviews were conducted with 4/18 ‘predominantly rural’, 4/9 ‘predominantly urban’ and 2/3 ‘urban with significant rural’ LTLAs.

**Fig. 1:**
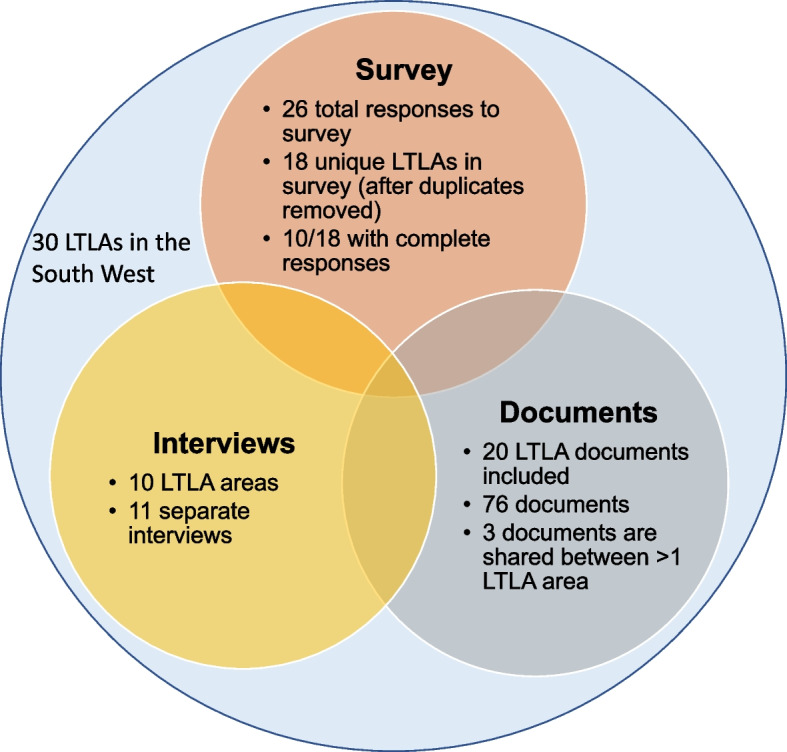
Summary of data sources included in evaluation

Of the 30 LTLAs, 22 had at least one data source included in the study and six were represented by all three sources (Fig. 1).

### Data analysis

Prior to coding, all transcripts, survey responses, and documents were read to gain a contextual understanding of the data. Survey data were exported from Qualtrics, cleaned, and merged with national Census data to generate a complete list of 30 LTLAs in the SW, including their three-fold Rural Urban Classification [28]. The number of PRS households (“private landlord or letting agency” or “other private rented”) and total households were downloaded from the 2021 Census [29]. Graphs were produced in Microsoft Excel.

All data were imported into Nvivo (v20.6 QSR International) to be coded. Each piece of data was stored as an individual file and each row of the survey generated a ‘case’ for all 30 LTLAs. Relevant interview and documents for that LTLA were added as sub-folders to this case, and coding of each case was aggregated from ‘children’.

Consistent with realist approaches, data analysis was retroductive [24]. The themes in the LGA toolkit were used as deductive mechanism codes, but further inductive contexts and mechanisms were coded when observed in the data. All analysis in NVivo was completed by CFF with reflective notes from ET to aid interpretation of the data. Initial familiarisation with the data was conducted by comparing contexts and mechanisms between LTLAs to look for patterns and evidence of IPTs. A memo was developed for each IPT using the template described by Gilmore et al*.* (2019) [26] so that each time an observable “context-mechanism-outcome” was found in the data source, this was re-coded and linked to the relevant IPT and the following headings of the memo were reviewed: context, mechanism, outcome, potential ICMOC, supports/ refutes/ refines, how/ why/ decision-making process, links to other IPTs and additional notes. A description of the type and level of action of mechanisms was included, as described by Westhorp in 2018 [30]. Refinement of IPTs occurred as additional data sources were coded and linked to the associated memos and through reviews with the research team (Table 1).

### Ethics and rigor

Ethics approval for this study was granted by Health and Applied Science Research Ethics Committee at UWE; Reference number HAS.22.06.128 on 15th July 2022. Informed consent was obtained from all subjects. An anonymous survey ID was generated to link survey data with interviewee respondents. All data was stored securely and only accessible by members of the research team.

To evidence transparency and rigor in the research approach, the RAMESES II reporting standards checklist has been completed [24] (Additional File 3).

## Results

This section commences with a discussion of the PRS housing market and the structure of the PRS team working in each LTLA. It then describes eight proposed IPTs to provide a nuanced view of the different mechanisms and associative conditions which underpin local government interventions to regulate the PRS within specific contexts. “If [Context], then [Outcome]. This is because [Mechanism]…" statements and a table describing the ICMOC for each proposed IPT are provided.

A key overarching finding is that there is a lack of objective outcomes being used to understand the nature and extent of impact of different mechanisms. There was evidence of softer outcomes such as improved relationships with colleagues, landlords, and tenants, and process measures using performance data such as the number of notices served, but the translation of this into positive impacts on PRS quality or health outcomes was absent. This hinders interpretation of the effectiveness of mechanisms in improving tenant health and wellbeing.

### Characteristics of PRS teams and stock

**Fig. 2:**
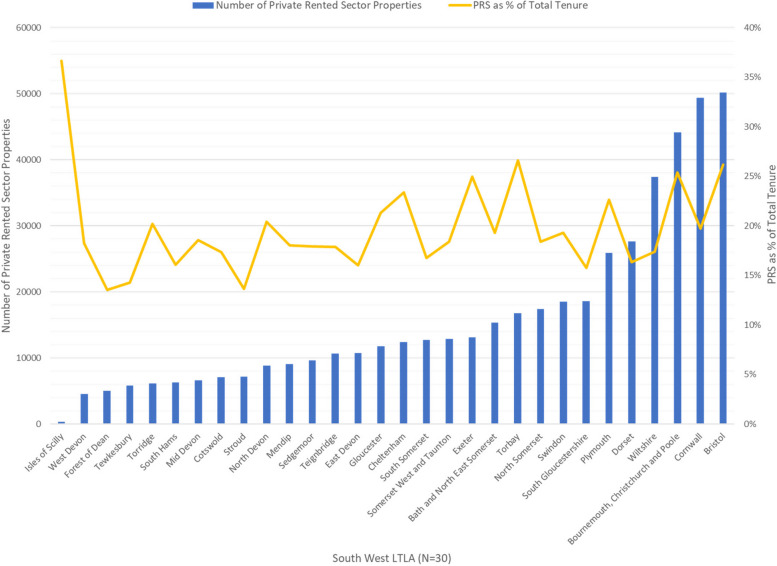
Number and proportion of Private Rented Sector properties by local government

There was variation across the SW in number of PRS properties and PRS housing as a proportion of total housing (Fig. 2).

Of the 10 LTLAs with complete surveys; the number of FTE staff working on the PRS varied from 4 to 40, and the number of PRS properties per FTE staff varied from 479 to 2880, with a median of 2121, which is in-line with national figures [31]. There was no clear association between percentage of PRS stock and PRS to staff ratio. The 10 interviews were conducted in LTLAs with medium to large numbers of PRS properties and medium PRS to staff ratios.

### IPT1—Belief in market forces

This first IPT captures how housing officers perceive their role in the PRS in the context of the wider housing crisis and want to improve standards without having a deleterious effect on the PRS market. The mechanism here is a subtle and often implicit belief in the natural balance of market forces to bring about positive change i.e. competition increases quality and keeps rental price low, resulting in a reluctance to interfere with market forces. There is evidence that PRS team leaders escalate concerns around housing supply to senior leaders and local politicians who, in turn, also consider market forces when making decisions.

Some interviewees suggested changes in legislation has forced landlords out of the sector and has led to, for example, an increased number of Airbnb’s. This reduces availability of properties in the PRS and/or increases rental costs for long-term tenants. The PRS team see their role as providing clear messages to landlords about local enforcement approaches but are conscious of setting thresholds which inadvertently drive them out, especially accidental or smaller portfolio landlords for whom profit margins may be tighter and inadvertent non-compliance more common.

*“You're always gonna get a percentage of properties that fall short… But if you're in a market that's, you know, pretty buoyant and the private rental sector is pretty buoyant. Then there was some sort of, there's some competition from landlords to deliver a reasonable service. So, we haven't got any market failure here. So, it's not gonna be impact in the same way, somewhere Liverpool, Humberside where they've had decades of market failure.” [Participant 7]*

This mechanism is embedded within the context of neo-liberal capitalism where free market principles favour reduced government spending, deregulation and privatisation. Even where officers demonstrate a hesitancy that this model is ineffective in progressing quality and equity in the PRS, there is reluctance for housing officers to completely disregard market dynamics. Yet, there may be more incentive to work with housing development teams to improve supply or enact alternatives to enforcement activities (see IPT4).

### IPT1 summary (Table 2)

**Table 2:** Intervention-Context-Mechanism-Outcome Configuration for Initial Programme Theory 1

Interventions	Context	Mechanism	Outcome
PRS team highlight impact legislation has on PRS market	Neo-liberal capitalism and degree of market successes and failures locally	Belief (to greater or lesser extent) in natural balance of market forces to bring about positive change i.e. competition increases quality and keeps price low, resulting in a reluctance to interfere with market-forces	The market primarily drives availability, affordability and quality of housing. In areas of market failure, additional interventions to improve PRS quality may be required but always within the context of market forces
PRS team work with housing options, homelessness prevention, housing development teams re supply			
Escalate concerns about PRS availability and affordability to senior leaders			

If there is no evidence of market failure, then the market primarily drives availability, affordability and quality of housing in the PRS. This is because there is an implicit belief in the natural balance of market forces bringing about positive change. If there is evidence of market failure, often in inner-city areas and in part due to demand outstripping supply (for example, from university students, economic migrants, refugee resettlement programmes or asylum seekers) and pressures from the cost-of-living crisis where rent represents an unaffordable proportion of income, then the PRS team are more likely to intervene to improve PRS quality. This is because the belief in market forces (alone) is insufficient to bring about positive change, but any interventions are still delivered within a context of not wanting to inadvertently undermine market forces.

### IPT2—Risk adverse to legal challenge

Another IPT, linked to market forces, is based on the perception of LTLAs to be risk adverse in their approach to managing the PRS. This is in reference to the requirement of LTLAs to provide strong evidence to bring about enforcement action, either at the level of an individual property relating to tenant eviction or in introducing area-wide interventions such as selective or additional licensing measures, that will often be highly unpopular with landlords (due to restricting market principles) and may lead to legal challenge. The context driving this risk-adverse mechanism is set by poor quality data and data systems resulting in difficulty evidencing the rationale for stronger enforcement activity, particularly if taken to court over these decisions and resource to fund expensive legal challenges is limited. This limits LTLAs from adopting hard enforcement interventions. The LTLA is perceived to have limited ‘power’ in changing or introducing regulation which may ultimately impact market forces.

*“If the Article 4 prevents unused houses or suitable houses being let as houses of multiple occupation, then that is blocking the opportunities that are available to help give move-on accommodation for people and I've highlighted that to our senior managers and they've taken it on board and said that, although they may not ultimately have the power to remove that restriction, it's definitely an area they want to consider because you have almost a left hand blocking the right hand there when you're trying to look for a solution.” [Participant 3]*

This next quote refers to an example of a team leader wanting to share a video on how to manage damp and mould in the PRS. It describes the risk-adverse culture in relation to social media use, but this was apparent in other aspects of regulating the PRS too.

*“The public sector is not as comfortable or good at using social media as maybe the private sector world is. And the level of, kind of, restriction or bureaucracy in terms of “let's hope we don't say the wrong thing”. So, everything that needs to be said has to go through so many checks and processes. Umm, for fear of maybe potentially saying the wrong thing or not communicating it in the best way possible.” [Participant 3]*

This mechanism is an example where regulations have the potential to force change and yet because of their complexity and the risk-adversity of LTLAs, due to poor data, these are less effective due to landlords remaining in control of the market. This relates to the next IPT which considers the LTLA’s attitude to enforcement.

### IPT2 summary (Table 3)

**Table 3:** Intervention-Context-Mechanism-Outcome Configuration for Initial Programme Theory 2

Interventions	Context	Mechanism	Outcome
Enforcement activity. E.g. Challenging Section 21 evictions, additional licensing, selective licensing	Poor quality data and data systems results in difficulty evidencing lack of improvements following enforcement or other interventions	LTLAs are risk adverse to intervening in the PRS due to the expensive and lengthy legal challenges that may result which resource-poor LTLAs are unable to withstand	Landlords remain in control of the market and exert power over LTLAs, preventing additional hard enforcement or other restrictive measures being implemented

If poor quality data and data systems result in difficulty evidencing lack of improvements following enforcement or other interventions, then landlords remain in control of the market and exert power over the LTLA’s decisions to limit market forces (for example, slow progress on individual enforcement cases and/or area-wide interventions such as introducing additional or selective licensing). This is because LTLAs are risk adverse in intervening in the PRS due to the risk of expensive and lengthy legal challenges which resource-poor LTLAs are unable to withstand, and this results in a perceived lack of power to regulate the sector.

### IPT3—Attitude to enforcement

This IPT suggests there are an array of factors associated with the philosophy of a local government as to how they govern PRS housing. Formal or hard enforcement approaches are often justified by quoting their 'statutory duty' to improve standards or assess risks and this was often influenced by previous experiences of the PRS team leader and/or local political support. Three LTLAs reported taking a harder approach to enforcement than in the past, one specified taking a softer approach, and the remaining were not explicit. Opinions varied as to whether hard enforcement was effective. Some LTLAs believed it encourages compliance and more efficient ways of working, whilst others felt it improved awareness but resulted in an increased workload. Current enforcement legislation is complex, costly and time-consuming to enforce, so often only the worst quality housing is improved to a minimum standard.

*“Well, I've always been brought up actually, if you want to put it that way is that taking formal approach is a better way of doing things than maybe what informal is. So it's just the way that I think that I've been brought up through my posts and through other managers that have managed me… on a personal note, I find it quite infuriating and frustrating when you get landlords that just don't understand the rules or don't want to play by the book. Especially in some of the conditions that we see and I think that that just touches a soft spot.” [Participant 1]*

The mechanism identified here is a reasoned psychological or cognitive approach at the individual PRS leader-level about the implicit appropriateness of taking a formal approach. This may extend to a type of ‘group-think’ at the PRS team or LTLA-level where formal enforcement is assumed preferable. As highlighted in the introduction to the results section, there is absence of evidence to evaluate whether formal enforcement drives up quality of the PRS and improves the health and wellbeing of tenants. As a result, the adopted approach is often driven by ‘gut feel’ rather than data or evidence.

### IPT3 summary (Table 4)

**Table 4:** Intervention-Context-Mechanism-Outcome Configuration for Initial Programme Theory 3

Intervention	Context	Mechanism	Outcome
Hard enforcement	Team leaders’ ‘upbringing’ and previous experience set expectations for attitudes to enforcement	A general perception that formal, hard enforcement is the ‘right’ way to do things at an individual, team or LTLA-level	Hard enforcement approaches prioritised regardless of outcomes / impact, a belief that hard enforcement brings about positive change

If PRS team leaders have previous experience of using hard enforcement approaches which may be influenced by personal preference and belief, then these will dominate intervention activities to improve quality in the PRS. This is because there is a general perception (irrespective of a lack of outcome data) that formal, hard enforcement is the ‘right’ way to do things.

### IPT4—Relational approaches to partnership working

This IPT is evidence that some housing officers understand and demonstrate compassion towards the vulnerability of ‘silent’ tenants, recognising their relative powerlessness to speak up and complain to landlords about poor housing conditions if the potential consequences include rent increases, eviction and homelessness. This leads to a belief in the importance and ultimate effectiveness of building trusting relationships with landlords and other partners, putting tenants’ wellbeing at the centre of what they are doing. These relational approaches include improving education and engagement, sharing good practice, supporting tenant groups, introducing landlord forums, accreditation schemes or star-rating systems, and establishing referral pathways between partners. In the LGA toolkit, these are referred to as ‘consumer regulation’, a term poorly understood by interviewees. Examples of educational activities included providing tenant and community resources on LTLA websites, YouTube or social media about the links between housing and health, how to reduce the risk of damp and mould, and information about rights and responsibilities.

*“[Re housing strategy] … It's mainly focused on landlords within the private rented sector to try to incentivise those improvements and link it with a referral scheme that they will take tenants from our homelessness service”. [Participant 10]*

This mechanism is in action in contexts where there is individual or organisational acknowledgement of the limitations of enforcement activities. It appears to be in opposition to the mechanisms described in IPT1 (belief in market-forces) and IPT3 (attitude to enforcement), although in reality, most LTLAs implement a mixture of formal enforcement alongside these relational approaches, acknowledging that enforcement may improve the worst quality housing within the PRS, but the vulnerable ‘silent’ tenant means relational approaches are also valued. It would also be incorrect to conflate market-driven forces with only enforcement activities since these alternative interventions may also have (although potentially less significant) influence on the housing market.

### IPT4 summary (Table 5)

**Table 5:** Intervention-Context-Mechanism-Outcome Configuration for Initial Programme Theory 4

Interventions	Context	Mechanism	Outcomes
Alternatives to enforcement	Individual or LTLA level acknowledgement of limitations of enforcement activities	Housing officers understand the vulnerability of ‘silent’ tenants. A belief in the importance and ultimate effectiveness of building trusting relationships with landlords and other partners, putting tenants’ wellbeing at the centre of what they are doing	Less dependency on hard enforcement
E.g. education and engagement, sharing good practice, supporting tenant groups, introducing landlord forums, accreditation schemes or star-rating systems, establishing referral pathways between partners			
			Limited evidence of outcomes (mixed positive/negative)

If PRS teams, and LTLAs more widely, acknowledge the limitations of formal enforcement activities to improve quality of PRS housing, then there will be less dependency on hard enforcement and alternative relational approaches will also be used. This is because housing officers understand the vulnerability of ‘silent’ tenants and believe in the importance and ultimate effectiveness of building trusting relationships with landlords and other partners, putting tenants’ wellbeing at the centre of what they are doing.

### IPT5—Job security and remuneration

Every interviewee cited insufficient resource limits their ability to improve quality in the PRS due to the context of budget constraints within local authorities. Several LTLAs reported this extended to insufficient funding to offer competitive salaries and recruit to permanent positions within PRS teams. This revealed an individual-level mechanism, based on the liability of job insecurity, to drive investment in the PRS team. External funding sources may, in turn, generate a wider scope of practice within the PRS team e.g. green initiatives or levelling up funding, generating a feed-forward loop increasing the scope of new funding opportunities. Equally, the dependency on grant-funding results in a risk of team turnover if grant funding is not received, or difficulty in recruiting permanent staff if there is no corporate pressure to improve low wages which remain uncompetitive.

*“We don't have the money to get more staff. We've got restrictions there and you know government are cutting funding as it is. So you know, as soon as there's any grant money from governments we’re there applying for it.” [Participant 1]*

This also drives innovative use of data to enable grant applications and further investment in the PRS as evidenced by the following quote:

*“I've just been working on looking at licensed HMOs and unlicensed HMOs and more to procuring more data from a data warehouse company which is property specific data… We will be using GIS and I'm going to be looking at using data much more to look at heat mapping, hotspot mapping, looking at potentially over layering health data, GP service data and then seeing what it shows us. I think getting the data is the hardest part and… why people don't like doing it is because it shows massive gaps in what they do with their own data and people don't like that being publicly disclosed, they're like, hang on that doesn't represent all the work we do.” [Participant 6]*

### IPT5 summary (Table 6)

**Table 6:** Intervention-Context-Mechanism-Outcome Configuration for Initial Programme Theory 5

Intervention	Context	Mechanism	Outcomes
External grants bring in additional funds to the PRS team	In the context of resource-limited LTLAs, PRS teams may be dependent on external grant-funded temporary staff due to low wages remaining uncompetitive	Self-determination and personal drive for job security and renumeration motivates PRS team leaders to seek additional funds for the team	This will likely create additional capacity and motivation within the team to do horizon scanning, grant applications and build partnership working where there may be more opportunity to hear about funding opportunities
	Larger LTLAs may have more capacity, cross-LTLA specialist support, and innovative data use to be more likely to apply for (and be successful in receiving) external grant funding		
			It also incentivises innovative use of data to demonstrate the demand for investment in the PRS

If team leaders are contracted on a temporary basis through externally funded grants, then this will likely create additional capacity and motivation within the team to do horizon scanning, grant applications and build partnership working where there may be more opportunity to hear about funding opportunities. It also incentivises innovative use of data to demonstrate the demand for investment in the PRS. Larger LTLAs, where there is greater cross-LTLA capacity from specialist teams in digital, comms and staff training, facilitate innovative data use including introducing digital platforms and complex data merging. This is because self-determination and personal drive for job security and renumeration, in the context of resource-limited LTLAs, motivates PRS team leaders to seek additional funds for the team.

### IPT6—Financial incentives drive action

A second IPT emerges here within the context of resource-poor LTLAs; there was consistent evidence of local and national sustainability targets and associated funding schemes driving PRS teams’ activities to increase awareness and incentivise landlords (and owner occupiers) to retrofit their housing stock and improve housing quality. The motivation here is the financial incentives associated with these new regulations for the landlord rather than an overt recognition of the risk of climate change directly. This is a good example of regulations acting as a ‘force’ mechanism at the organisational-level [30].

PRS leaders acknowledge the risk of increased damp and mould if retrofitting inadvertently worsen ventilation, depending on the tenant’s behaviour and usage of the property. They also acknowledged that the renovation works could be disruptive to tenants but the benefit of reducing utility bills usually outweighed the disruption.

### IPT6 summary (Table 7)

**Table 7:** Intervention-Context-Mechanism-Outcome Configuration for Initial Programme Theory 6

Interventions	Context	Mechanism	Outcome
Domestic Minimum Energy Efficiency Standard Regulations and requirement to have an Energy Performance Certificate (EPC)	Number and availability of green incentives for improving the EPC rating of housing stock, including the PRS	Resource limitation to invest in the PRS in general, means that financial incentives have driven action in this area	Retrofit housing to improve insulation, reduce utility bills, and pressure on finances for people living in poverty leading to warmer homes with better health outcomes
	Universal context of resource-limited LTLAs		
Green grant funding initiatives			

If green funding incentives are available to LTLAs, PRS teams will seize the opportunity given otherwise resource-limitations, to make grants available to landlords (and owner occupiers) to retrofit housing and improve insulation, consequentially leading to warmer, healthier homes. This is because the financial incentives, both in terms of getting insulation installed (for landlords) and reduction in utility bills (for tenants) are driving action in this area.

### IPT7—System level understanding of the drivers of poor health and inequalities

The final two IPTs are about system-level working where effective strategic direction has been set by a wide range of organisations and stakeholders. The understanding of the drivers of poor health and inequalities is a feed-forward looping mechanism, reinforcing the benefits of partnership working. Several LTLAs were trying to merge datasets such as LTLA tax, parking penalties, and Energy Performance Certificates (EPCs), to identify PRS properties and prioritise the poorest quality ones for improvements but frequent challenges with poor data quality and authorisation to share data limited the effectiveness of this.

*“There's a drive within [the LTLA] to try and identify, digitally identify their customers, and in place to where they are and who they are for, how they can then engage with them productively… so they're working hard to cross match various data sets using algorithms probably to predict whether something's PRS or not”. [Participant 5]*

This mechanism is apparent in areas with a visible concentration of high-density poor-quality PRS housing such as inner-city areas of deprivation and/or where there is a high proportion of university student housing. There was no evidence of this mechanism in rural areas where the PRS is poorly identified and there is not the same opportunity to draw attention to the issue.

*“...actually we're getting much better at working together and sharing information. So being aware of what each of us do is being really positive... there's a lot of multi-agency coordinated projects and forums, so we've got the housing, health and care partnership... there was a report produced with [the Director of Public Health], who produced it all around health inequalities in [the local area], and housing was one of the key outcomes of that. That is, that's where the biggest disparity sits. So there's lots of, I think, positive meetings and conversations that are happening now.” [Participant 6]*

### IPT7 summary (Table 8):

**Table 8:** Intervention-Context-Mechanism-Outcome Configuration for Initial Programme Theory 7

Interventions	Context	Mechanism	Outcome
PRS escalate concerns about poor quality PRS so that system partners are aware of issue Partnership working	Visible concentration of high-density poor-quality PRS, often in inner-city areas and/or where there is a high concentration of university students	System level understanding of the drivers of poor health and inequalities drives strategic vision and investment in the PRS team and wider partnership working	Prioritisation of resource and investment in the PRS team and wider partnership working to improve the quality of the PRS, and subsequently the health and wellbeing of tenants and wider social issues

If there is a visible concentration of high-density poor-quality PRS housing, then this aids resource prioritisation and investment in the PRS team and wider partnership working with an aim of improving the quality of the PRS. The potential benefits of improving PRS stock are noted to extend beyond improving the poor health of tenants to addressing wider local issues such as crime, antisocial behaviour, illicit drug markets, waste management and zero carbon commitments. This is because the system-level understanding of the drivers of poor health and inequalities, and the vulnerability of tenants living in poor quality housing made visible through partnership working, drives strategic vision and prioritisation of this sector.

### IPT8—Understanding of cost-saving opportunities

This mechanism, strengthened by partnership working specifically with adult social care, is about highlighting and understanding cost-saving opportunities of early intervention in an ageing population living in the PRS to avoid high social care costs.

### IPT8 summary (Table 9):

**Table 9:** Intervention-Context-Mechanism-Outcome Configuration for Initial Programme Theory 8

Intervention	Context	Mechanism	Outcome
The use of Disabled Facilities Grants (DFG) to adapt homes so people can stay independent for longer	Ageing population living in PRS accommodation Very high social care budgets in local authorities	System level understanding of the drivers of poor health, inequalities and tight LTLA budgets drives strategic vision and investment in the PRS team and wider partnership working	Home adaptations facilitating people to stay in their PRS accommodation to reduce social care costs

If there is an ageing population and high-cost burden associated with social care, then home adaptations facilitating people to stay in their own home and remain independent, preventing hospital and/or social care admissions, will be overall cost-saving. The demand for appropriately adapted housing in current and prospective housing stock is well-understood. As the PRS-specific population is also ageing, the benefit of working with landlords to facilitate these adaptations is increasingly recognised. For example, PRS team leaders promote the use of DFGs with landlords. This is because this system-level understanding of the drivers of poor health, inequalities and tight LTLA budgets drives strategic vision and investment in partnership working across the system (e.g. between adult social care, occupational health and housing).

## Discussion

This study adds value to the evidence base by using a realist framework to understand which factors influence the development and choice of interventions in local government to manage the quality of PRS housing and therefore the health and wellbeing of tenants. The findings suggest that the mechanisms that bring about a positive outcome in managing the PRS are unlikely to be universal; they depend on the context which differs across place and over time. This highlights the need for strategies to be tailored considering the local context. It provides a starting point for researchers in the field to test these plausible hypotheses to refine and deepen our understanding of how PRS housing interventions which are beneficial for tenants' health can be delivered. A key strength of this study was the multi-step, mixed method approach, which incorporates numerous sources of evidence to iteratively produce robust IPTs. Future work should now seek to refine and expand these IPTs through further testing in other parts of the UK and internationally, with the aim of reaching a Middle Range Theory [24, 26].

The findings yielded eight IPTs which act on different levels from individual PRS team leaders to organisational and across the system. The IPTs include a belief in market forces, risk adversity to legal challenge, attitude to enforcement, relational approaches to partnership working, job security and renumeration, financial incentives drive action, and system-level understanding of the drivers of poor health, inequalities and opportunities for cost-savings. Importantly, the lack of objective outcomes being used to understand the nature and extent of impact different mechanisms were having on PRS quality and the health and wellbeing of tenants’, hinders interpretation of the effectiveness of all mechanisms. Several of these mechanisms (particularly IPT1, 3 and 4) are examples of where the cultural background of the individual has shaped the process by which they acquire and interpret information. This is described as ‘cultural cognition’ referring to a ‘tendency of individuals to confirm their beliefs around disputed matters of fact’ particularly apparent in this sector where there is a paucity of outcome data [32]. The relation between these IPTs is not always apparent, due to the differing contexts, yet the system-level IPTs (7 and 8) are likely to be assimilated from multiple, smaller, contributing mechanisms described in IPTs 1–6, which shape individual and organisational values and a system-wide culture of continual learning about the PRS locally and the health and wellbeing of tenants.

Some of these contexts and mechanisms were already known. For example, there has long been debate about the optimal enforcement approach. Our finding of mixed views towards hard enforcement are consistent with earlier studies [16, 33]. Similarly shifts in demand on PRS [17], and the significant lack of resource and capacity within the public sector [34, 35] have been previously described. Interesting null findings included that; unitary compared to two-tier authorities, the local government department which the PRS team sits within, and the understanding of the wider determinants of health of the housing officer were not found to have an impact on the ability to utilise mechanisms.

The lack of suitable outcome measures has been reported by other studies, whereby local governments tend to blur activity or process measures (e.g. number of prosecutions) with outcome measures (e.g. number of properties improved) [16]. This is a critical issue because it limits the ability to reach consensus within the sector on what mechanisms are effective and how best to target the use of limited resources. This suggests increased support for local governments to understand the potential datasets available, and ways to merge them would be valuable. It is recommended that in addition to housing stock condition databases and deprivation data, that objective measures from local public health and healthcare datasets are used. This could enable better identification of vulnerable households within the PRS and therefore targeting of limited resources. The use of tools like the Housing Health Cost Calculator [36], which quantifies the extent to which improvements in housing can reduce pressure on health services, could help make the consequences of poor quality PRS more visible. Although many of the factors which affect the demand for PRS housing are not within the control of local governments, data can be useful to monitor and predict the impact on tenants and their needs. For example, the rising cost of living that many high-income countries are experiencing is having a greater impact on people living in the PRS than other housing, with two in five renters finding it difficult to pay their rents, compared to one in five homeowners [37]. The recognition of cultural cognitive bias in this study requires a conscious effort and openness for LTLAs and local systems to learn from other local areas and abroad. For example, home ownership is valued very differently in some other European countries [38].

Given the finding that meeting environmental sustainability targets was seen as a potential driver for incentivising improvements to PRS housing, it is important to understand the interplay between climate and health agendas, such as health outcomes associated with energy efficiency interventions, e.g. increased insulation and reduced ventilation, as promoted widely during the Cost of Living Crisis.

There continues to be much regulatory change affecting PRS housing. These changes will have complex impacts and take time to emerge [17]. As this evaluation was conducted at a similar time to the publication of ‘A fairer private rented sector’ White Paper [18], there will be upcoming opportunities to evaluate additional mechanisms available to local government. If the Renters Reform Bill is passed, local governments will have more power to enforce and protect tenants’ rights, including a register of landlords and end to ‘no fault’ evictions [18]. Importantly, a new Decent Homes Standard may put social and PRS housing on the same level in terms of regulatory expectations [39] and there are new regulations to remove the requirement for accommodation for asylum-seekers provided on behalf of the Home Office to have an HMO license from local government (The Houses in Multiple Occupation (Asylum-Seeker Accommodation) (England) Regulations, 2023) [40]. Finally, the relevance of referral pathways (an intervention identified within IPT4) has increased following a recent landmark case of a child’s death where housing conditions were held directly responsible [41]. Health and social care teams referring priority patients with health needs for better housing conditions, could therefore become a more prominent mechanism in the future [42].

The findings of this research illustrate the importance of considering the different contexts within which new local government regulatory powers and responsibilities will be applied. The effectiveness of the Government’s plans for reforming the PRS in England, will crucially depend on the extent to which local authorities are able and willing to apply the legislation in practice. The findings demonstrate that although resources are a key determining factor, they are by no means the only driver. A full consideration of the range of factors which influence the way in which the sector is regulated at a local level, should be an integral part of any impact assessment of the new regulation.

### Strengths, limitations and future research

As with all realist evaluations, this study is inherently interpretative. The elicitation of ICMOCs and the refinement of theories has been dependent on the researcher teams’ judgment and existing knowledge introducing a possibility of bias [26, 43]. Care has been taken to document in detail the “decision-making” processes within the analysis, to help to ensure transparency across this evaluation.

As the interviews were conducted by public health professionals, it is possible this led to reporting bias, with participants overemphasising their understanding of ability to influence health. Given the notable discrepancies between duplicate responses from different cadre of staff, for example on estimates of staff working within the PRS team, participant bias and the reliability of participant responses could be questioned. Using the research teams’ professional experience in order to guide participant selection hopes to have captured innovative work in the PRS, however it may have also led to some selection bias, with housing teams more engaged in the health agenda being chosen. To minimise the extent of these biases, a high degree of rigor has been taken, as evidence by the RAMESES II checklist (Additional File 3).

The research was conducted in one geographical region, and whilst this is fairly representative of much of England, there are notable policy differences across the wider UK and internationally [17]. Despite this, we propose that our findings could be generalisable to the wider UK and other countries, due to the breadth of local government structures, sizes, staffing, and interventions used, which were included in the study. Many of the contexts and mechanisms that were present in this evaluation would apply to other countries, for example increasing demand on PRS from population changes and the Cost of Living crisis.

## Conclusion

To our knowledge, this evaluation is the first to use realist methodology to examine factors which influence local government interventions for managing the quality of PRS housing to improve the health and wellbeing of tenants. This allowed identification of the extent to which different mechanisms are being used, and, crucially, the different contextual factors which affect this. Eight new IPTs about what works, for whom, under what circumstances have been developed. The findings are not only theoretically novel, but also have practical relevance for those developing and delivering new interventions on housing and health, and providing recommendations on how to optimise, tailor, and implement, existing mechanisms and design and measure outcomes to monitor improvements. These will be particularly relevant for academic researchers, and housing and public health professionals, especially those working in local governments.

### Supplementary Information

Supplementary Material 1.

Supplementary Material 2.

Supplementary Material 3.

## Data Availability

The datasets used and/or analysed during the current study are available from the corresponding author on reasonable request.

## Abbreviations

DLUHC: Department for Levelling Up, Housing and Communities
EPC: Energy Performance Certificates
FTE: Full-Time Equivalent [staff]
HHSRS: Housing Health and Safety Rating System
HMO: Houses of Multiple Occupation
ICMOCs: Intervention-context-mechanism-outcome configurations
IPT: Initial Programme Theory/ies
LGA: Local Government Association
LTLA: Lower-Tier Local Authorities
MEES: Minimum Energy Efficiency Standard
NHS: National Health Service
ONS: Office of National Statistics
PRS: Private Rented Sector
SW: South West
UK: United Kingdom
UWE: University of the West of England
