# Supplementary material for: Local government interventions for improving the health and wellbeing of tenants in private rented housing: developing initial program theory to inform evaluation in the United Kingdom
Source: BMC Public Health. 2024 Aug 7;24:2144. doi: 10.1186/s12889-024-19163-9 (PMC11308383; doi:10.1186/s12889-024-19163-9)
Supplement: Supplementary file 1 — Supplementary Material 1. [file 12889_2024_19163_MOESM1_ESM.docx]

**Additional File 1: Survey**

3.1 ABOUT YOU

3.2 Job Title

________________________________________________________________

3.3 What aspects of private rented sector housing work do you have responsibilities for?

- Policy  (1)
- Partnership  (2)
- Enforcement  (3)
- Managerial  (4)
- Strategic  (5)
- Homelessness prevention  (6)
- Other  (7) __________________________________________________

3.4 Which local authority or district do you work for? 

________________________________________________________________

3.5 Is this local authority or district predominantly:

- Urban  (1)
- Rural  (2)
- Both  (3)

3.6 How many FTE employees work in the PRS housing team of your local authority / district excluding the Housing Service lead?  
 
Please include all staff working in the PRS housing team including administrative support, enforcement staff, team leaders, head of PRS department.

________________________________________________________________

4.1 EVIDENCE BASE

4.2 Have you up-to-date and accurate information on the overall size and condition of the private rented sector?

- Yes  (1)
- No  (2)

4.3 What is the estimated size of the PRS stock?

- Estimate size:  (1) __________________________
- Don't know  (2)

4.4 What is this estimate based on?

________________________________________________________________

4.5 Have you a timetable in place to improve your evidence base (eg stock condition surveys and environmental performance) and that takes account of the 2021 Census results?

- Yes  (1)
- No  (2)

4.6 Do you have information on and/or understanding of the changing pattern of demands among different groups of tenants?

- Yes  (1)
- No  (2)

4.7 Do you have information on and/or understanding of any changes in the types of landlords and lettings agents operating in your area?

- Yes  (1)
- No  (2)

4.8 Do you have information on and/or understanding of the different types of private rented stock in your area eg houses in multiple occupation (HMOs), traditional family accommodation, purpose-built student accommodation and build-to-rent schemes?

- Yes  (1)
- No  (2)

4.9 Do you bring together and analyse relevant internal databases to improve your evidence base eg council tax records, planning applications and enforcement action?

- Yes  (1)
- No  (2)

4.10 Do you effectively collaborate with any of the following to help identify patterns of poor health and vulnerability within the private rented sector?

- Adult social care  (1)
- Children's services  (2)
- Public Health teams  (3)
- Healthcare services  (4)
- Third Sector Organisations  (5)
- Private consultancies  (6)
- Sub-regionally with other councils / districts  (7)
- Other  (8) __________________________________________________
- None  (9)
- Don't know  (10)

 4.11 Does your local authority / district analyse and understand the role of the private rented sector in relation to the overall housing market (eg owner occupation and social renting)?

- Yes  (1)
- No  (2)
- Don't know  (3)

4.12 Does your local authority / district have agreements in place to share and exchange information with other organisations (eg fire and rescue, DWP, HMRC, Border Force and Gangmasters and Labour Abuse Authority [GLAA])?

- Yes  (1)
- No  (2)
- Don't know  (3)

4.13 Do you review research publications on the private rented sector by organisations such as the UK Centre for Collaborative Housing Evidence (CaCHE)?

- Yes  (1)
- No  (2)

5.1 POLICY and POLICY MAKING

5.2 Do you have an up-to-date strategy for the private rented sector?

- Yes  (1)
- No  (2)

5.3 Do you evaluate the implementation of your PRS strategy using any of the following measures:

- Regulatory compliance  (1)
- Improvements to the housing stock outcomes  (2)
- Health and wellbeing outcomes  (3)
- Performance measures eg inspections, notices issued  (4)
- Other  (5) __________________________________________________
- None  (6)

5.4 When prioritising objectives in order to take account of resource constraints, is health taken into consideration?

- Yes  (1)
- No  (2)

5.5 In your opinion, is there an adequate balance between incentives and enforcement for landlords and letting agents?

- Yes  (1)
- No  (2)

5.6 Are your PRS strategy or policies aligned with

|  | Yes (1) | No (2) | Don't know (3) |
| --- | --- | --- | --- |
| Local authority / district strategies or plans, such as the local plan (including supplementary planning documents - SPDs), the housing strategy and the corporate plan? (1) |  |  |  |
| improving the environmental performance of the PRS stock in the light of the climate change emergency? (2) |  |  |  |
| other specific housing strategies, eg student housing? (3) |  |  |  |
| strategies on neighbourhood renewal including empty homes strategies? (4) |  |  |  |
| strategies to tackle modern slavery? (5) |  |  |  |
| strategies to tackle poverty and deprivation eg fuel poverty, council tax reductions? (6) |  |  |  |
| strategies to improve public health and wellbeing eg affordable warmth, trips/falls, overcrowding? (7) |  |  |  |
| strategies on homelessness and rough sleeping and the use of the PRS sector for temporary and permanent accommodation? (8) |  |  |  |

6.1 RESOURCES

6.2 Do you have the capacity to effectively respond to any external funding sources eg Department of Levelling Up, Housing and Communities (DLUHC) to support your work in the private rented sector?

- Yes  (1)
- No  (2)

6.3 Do you work with partners to maximise resources such as finance and skills?

- Yes  (1)
- No  (2)

7.1 GOVERNANCE

7.2 Are there effective links with other in-house services eg housing advice, homelessness, planning etc to achieve the wider aims and objectives of your housing strategy?

- Yes  (1)
- No  (2)

7.3 Is there a local councillor who advocates for improvements in the private rented sector effectively?

- Yes  (1)
- No  (2)

7.4 Are monitoring reports regularly presented to senior management teams and to cabinet/ committees?

- Yes  (1)
- No  (2)

8.1 PARTNERSHIPS 
You told us that you effectively collaborate with the following to help identify patterns of poor health and vulnerability:
 
Other key partners and stakeholders may include landlords’ associations, tenants’ groups, community organisations and universities.   

8.2 Are the objectives, requirements and views of partners and stakeholders understood and acted upon?

- Yes  (1)
- No  (2)

8.3 Are there regular meetings with partners and stakeholders at an officer level to discuss policy and operational issues?

- Yes  (1)
- No  (2)

9.1 LANDLORDS AND TENANTS SUPPORT

9.2 Is supporting landlords and tenants a priority in the strategy for the private rented sector?  

- Yes  (1)
- No  (2)

9.3 Do you operate or support a property management service / social lettings agency?

- Yes  (1)
- No  (2)

9.4 Do you support and encourage tenants’ groups?

- Yes  (1)
- No  (2)

9.5 Do you operate a landlords’ forum?

- Yes  (1)
- No  (2)

9.6 Do you operate or support a landlord accreditation scheme?

- Yes  (1)
- No  (2)

9.7 Do you operate or support a tenant advice service that covers the private rented sector?

- Yes  (1)
- No  (2)

9.8 Do you make use of or signpost initiatives such as ‘marks out of tenancy’ to tenants and landlords?

- Yes  (1)
- No  (2)

10.1 ENFORCEMENT

10.2 Do you operate a reactive-only enforcement policy based on complaints by tenants and communities?

- Yes  (1)
- No  (2)

10.3 Do you operate regulatory partnerships with any of the following agencies:

- Fire and Rescue  (1)
- Trading Standards  (2)
- Department of Work and Pensions  (3)
- HMRC  (4)
- Border Force  (5)
- Gangmasters  (6)
- Labour Abuse Authority (GLAA)  (7)
- Other  (8) __________________________________________________
- None of the above  (9)

10.4 Are short-term lets (eg Airbnb) a growing issue?

- Yes  (1)
- No  (2)

10.5 Have you considered the implications over the next decade of a growth of the number of older households in the private rented sector?

- Yes  (1)
- No  (2)

10.6 If relevant, are you working with universities to map out future student accommodation needs?

- Yes  (1)
- No  (2)
- Not Applicable  (3)

10.7 Are office conversions without the need for planning permission (ie permitted development) a growing issue?

- Yes  (1)
- No  (2)

10.8 Is the quality of ‘exempt accommodation’ (ie temporary accommodation) an increasing issue?

- Yes  (1)
- No  (2)

11.1 FINAL PAGE

11.2 Please complete the Captcha verification:

11.3 Here is your Survey ID number:
 
**Please make a note of this ID number** which you will need to provide if you wish to withdraw from the study within 30 days. 
We will also ask for this ID number if we approach you to take part in a follow-up interview which will allow us to link your survey responses. 
 
Once you've copied your ID number, click Submit to finish the survey.  
