# Supplementary material for: Local government interventions for improving the health and wellbeing of tenants in private rented housing: developing initial program theory to inform evaluation in the United Kingdom
Source: BMC Public Health. 2024 Aug 7;24:2144. doi: 10.1186/s12889-024-19163-9 (PMC11308383; doi:10.1186/s12889-024-19163-9)
Supplement: Supplementary file 2 — Supplementary Material 2. [file 12889_2024_19163_MOESM2_ESM.docx]

Additional file 2

.docx

Title: Interview guide

This additional file contains the interview topic guide used in in-depth interviews with participants.

**Additional File 2: Interview guide**

|  | **Research question** | **Question area** | **Prompt** |
| --- | --- | --- | --- |
| 1 | Personal rapport | Thanks and introduction - [Confirm information from survey] | Introduce self & research team/topic  Confirm consent (received by email prior to interview)  Some questions from the survey will be repeated but then we will expand on them.  Check documents shared so far – anything missing?  How their info will be used  Duration (45-60mins)  Reassurances – anonymity, confidentiality, collective results  Reminder about audio recording  Any questions/concerns  Press RECORD – then state Survey ID for recording |
| 2 | Personal rapport | What LA/district do you work for?  How long have you worked there?  Can you tell me a little about your current role? | Participant background / Build rapport – get them talking and at ease. |
| 3 | Personal rapport | Reflecting on your professional experience, what do you think are the wider factors which influences the health of people living in your area? | In Public Health we use the term ‘wider determinants of health’ to describe the range of factors which impact on people’s health and wellbeing; from a person’s individual characteristics and behaviours, to social and economic conditions, to the physical environment.  Housing officers often work across the wider determinants of health, for example to improve the quality of housing. Can you tell me what this means to you in your practice? |
| 4 | Health impacts | Is there anything distinct about private rental sector housing and it’s impact on the health and wellbeing of tenants? | Please give an example/ case study  Can you think of particular exposures? Eg. physical but also psychosocial (security, affordability) |
| 5 | Health impacts | [only ask if vulnerable people not discussed in question above]    Do you feel there are any people living in this local authority/in this area that are particularly vulnerable to the negative effects of private rented sector housing on health? | Please give an example/ case study  For example, lower income, in receipt of benefits, living with disabilities, etc |
| 6 | Use of mechanisms | What are the main approaches you adopt to improve the health and wellbeing of tenants in private rented housing? | If prompts are required, interviewer may highlight below themes as options:   1. Evidence base 2. Policy and policy making 3. Resources 4. Governance 5. Partnerships 6. Consumer regulation 7. Enforcement 8. Emerging issues |
| 7 | Contextual factors | What is limiting your ability to improve the health and wellbeing of tenants in the PRS locally? | Why?  Please give an example. |
| 8 | Contextual factors | What is working well currently and enabling you to improve the health and wellbeing of tenants in the PRS locally? | Why?  Please give an example. |
| 9 | Contextual factors | (Emerging issues)  The Government has proposed to introduce a number of new laws and regulations to aim to improve standards in the PRS. What impact, if any, do you think this will have in the local area?    Follow-up: are there any other emerging issues that you are aware of? | Prompt: How does this affect you locally?    Reminder of main proposals in white paper [if required]: Landlord register, Update to Decent Homes Standard applying to PRS, Removal of Section 21 evictions. |
| I’d like to ask about some of the different mechanisms which are available to Local Government to improve the quality of private rented housing, and therefore health and wellbeing of tenants… | | | |
| 10 | Contextual factors | (Evidence base)  How do you currently use data to inform your actions on private rented housing, and how could this be improved? | For example evaluations, stock surveys, rouge landlord database, mandatory register of landlords, financial data, administrative data from other departments, census, etc  Please give an example. |
| 11 | Contextual factors | (Policies and Strategies)  Can you tell me about your current housing policies or strategies – does it specify PRS as a sector? Do they have any links to other relevant health policies or strategies?    Follow-up Q: Conversely, do you know if local health strategies or policies link to housing docs? | Other relevant policies, may include, Health and Wellbeing strategy and JSNAs, homelessness, modern slavery, affordable warmth, etc |
| 12 | Contextual factors | (Partnerships)  What internal and external partnerships do you draw on to support work on private rented housing and how could this be improved? | Internal, eg. Local authority public health teams,  External, eg. health and social care (ICS/PCNs etc) VCSE, Sub-regional, fire and rescue, DWP, HMRC, Border Force and Gangmasters and Labour Abuse Authority [GLAA] |
| 13 | Contextual factors | Are there any other aspects of the survey which you would like to expand on which may have relevance to health? | Always prompt: Resources, Governance, Regulation, Enforcement |
| 15 | Close | Do you have any further comments?  Thank you for your time. | End Recording |
